# An Insert Goniometer Can Help Select the Optimal Insert Thickness When Performing Kinematically Aligned Total Knee Arthroplasty with a Medial 1:1 Ball-in-Socket and Lateral Flat Surface Insert and Posterior Cruciate Ligament Retention

**DOI:** 10.3390/bioengineering11090910

**Published:** 2024-09-12

**Authors:** Sahil A. Sanghavi, Alexander J. Nedopil, Stephen M. Howell, Maury L. Hull

**Affiliations:** 1 Department of Arthroplasty, Sancheti Institute for Orthopaedics and Rehabilitation, Pune 411005, India; 2 Department of Orthopaedic Surgery, König-Ludwig-Haus, University of Würzburg, Brettreichstr. 11, 97074 Würzburg, Germany; 3 Department of Biomedical Engineering, University of California at Davis, One Shields Avenue, Davis, CA 95616, USA; 4 Department of Biomedical Engineering, Department of Mechanical Engineering, Department of Orthopaedic Surgery, University of California at Davis, One Shields Avenue, Davis, CA 95616, USA

**Keywords:** total knee replacement, screw home mechanism, quadriceps line of force, tibiofemoral kinematics, internal tibial rotation, external tibial rotation

## Abstract

Current surgical practices in total knee arthroplasty (TKA) have advanced and include significant changes and improvements in alignment philosophies, femorotibial implant conformities, and ligament management to replicate in vivo knee kinematics. While corrective measures have emphasized sagittal plane alignment to restore normal flexion–extension (F–E) motion and coronal plane ligament balance, internal–external (I–E) rotation kinematics in the axial plane have been largely neglected. Recent in vivo evidence indicates that the combination of factors necessary to closely restore native tibial rotation as the knee flexes and extends is kinematic alignment (KA), which resurfaces the patient’s pre-arthritic knee without releasing ligaments, an insert with medial 1:1 ball-in-socket conformity and a lateral flat surface, and posterior cruciate ligament (PCL) retention. However, the inherent anterior–posterior (A–P) stability provided by the medial 1:1 ball-in-socket limits the surgeon’s ability to select the correct insert thickness using manual laxity testing. Accordingly, this review presents the design and validation of an instrument called an insert goniometer that measures I–E tibial rotation for inserts that differ in thickness by 1 mm and uses rotation limits at extension and 90° flexion to select the optimal insert thickness. The optimal thickness is the one that provides the greatest external tibial orientation in extension and internal tibial orientation at 90° flexion without lift-off of the insert.

## 1. Introduction

Contemporary surgical practice for total knee arthroplasty (TKA) has advanced to consider different alignment methods and implant designs to reproduce in vivo knee kinematics. While surgical techniques have long focused on coronal plane alignment to restore native flexion–extension (F–E) motion and coronal plane ligament balance, the internal–external (I–E) rotation kinematics in the axial plane have been largely ignored. Surgeons have a variety of femorotibial implant designs and tibial insert conformities to choose from, and there are differing opinions on whether to retain or excise the posterior cruciate ligament (PCL). According to American Joint Replacement Registry (AJRR) data, over 50% of primary TKA procedures performed until 2019 used posterior-stabilized (PS) designs. However, this trend has since reversed, with the 2023 AJRR data showing that cruciate-retaining (CR) designs accounted for 56.1% of primary TKA procedures in 2022. Despite the traditional popularity of PS and CR designs, medial-stabilized (MS) designs have shown better outcomes in comparison to CR and PS designs with kinematic alignment TKA [1,2].

**Figure 1:**
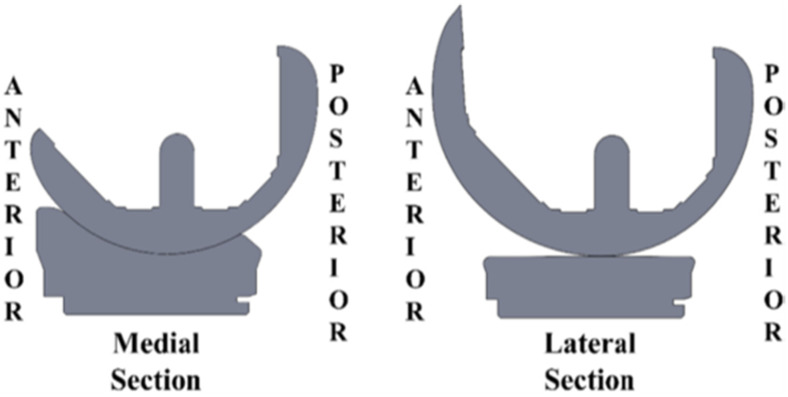
Section views of the femoral condyles and tibial insert in the A–P direction and through the dwell point of the medial 1:1 ball-in-socket insert showing articular geometry in the medial and lateral compartments.

This bioengineering review begins with an analysis of biomechanical studies of the native knee indicating that conditions for restoring native tibial rotation in flexion–extension of the knee include the combination of kinematic alignment (KA), which resurfaces the patient’s pre-arthritic knee without releasing collateral ligaments, an insert with a medial 1:1 ball-in-socket conformity and a lateral flat surface, and posterior cruciate ligament (PCL) retention (Figure 1). The lack of collateral ligament release is a profound advantage of KA over mechanical alignment (MA) where collateral ligaments are released in the majority of cases [3,4,5]. The advantage is profound because arguably most complications in MA are that the knee is unstable or too stiff, both of which are traced to incorrect collateral ligament release. Hence, these complications are largely avoided with KA.

A potential limitation of the inherent stability provided by the medial 1:1 ball-in-socket insert is that the surgeon loses the ability to select the correct insert thickness to balance the TKA using a manual assessment of anterior–posterior (A–P) laxity. Balancing the TKA is a critical intraoperative step because an insert that is too thin leads to instability [6], whereas an insert that is too thick causes stiffness, loss of motion, and high tibial contact forces [7]. To overcome this limitation and select the optimal insert thickness to properly balance the knee, this review introduces to the surgeon and bioengineer the design and validation of a novel instrument called an insert goniometer. This instrument measures internal–external (I–E) tibial orientation for inserts that differ in thickness by 1 mm and uses orientation limits at extension and 90° flexion to select to the optimal insert thickness. The optimal thickness is the one that simultaneously provides the greatest external tibial orientation in extension and internal tibial orientation at 90° flexion without lift-off of the insert. What the reader can expect to learn from this review is the following:

(i) The surgical alignment goal of KA and how to achieve it via proper balancing in the coronal and sagittal planes;
(ii) The biomechanical rationale for using the medial 1:1 ball-in-socket insert with a lateral flat surface;
(iii) The biomechanical rationale for retaining the PCL;
(iv) The benefits of KA for restoring normal patellar tracking;
(v) A description and an illustration of the insert goniometer and its compatibility with an insert with medial 1:1 ball-in-socket conformity and lateral flat surface;
(vi) An example describing and illustrating the use of the insert goniometer.

## 2. The Combination of Kinematic Alignment (KA), an Insert with Medial 1:1 Ball-in-Socket Conformity and a Lateral Flat Surface, and Posterior Cruciate Ligament (PCL) Retention Closely Restores In Vivo Native Knee Kinematics 

### 2.1. Surgical Alignment Goal of KA and How to Achieve It via Proper Balancing in the Coronal and Sagittal Planes

In TKA, the first step required to restore in vivo kinematics is to position the implants using caliper-verified KA. KA aims to resurface the patient’s pre-arthritic knee, which restores the native femoral and tibial joint lines and aligns the knee’s three kinematic axes with those of the femoral and tibial components contingent on the use of components that restore articular morphology and soft tissue constraints [7,8,9,10]. Thus, as it is a true resurfacing procedure, KA retains the native resting lengths of the collaterals and the PCL, thereby eliminating the need for their release [11]. 

KA resurfaces the patient’s pre-arthritic knee by matching the resection thickness of the distal femoral resections and the posterior femoral resections to the corresponding thickness of the distal and posterior condyles of the femoral component, after accounting for cartilage wear and the kerf of the saw blade. The caliper verifies that the thickness of each femoral resection is within 0 ± 0.5 mm of the target. 

**Figure 2:**
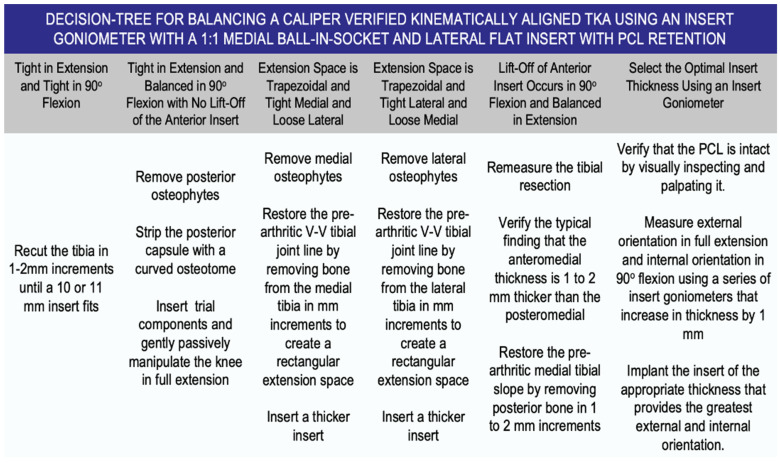
Composite showing the decision tree followed by the surgeon when he/she performs caliper-verified KA TKA. The technique sets the components to restore the pre-arthritic distal and posterior femoral and proximal tibial joint lines within 0 ± 0.5 mm, which restores native tibial compartment forces without the release of healthy ligaments, including the PCL.

The KA TKA is balanced by resecting the proximal tibia so that the varus–valgus (V–V) and flexion–extension (F–E) orientations of the tibial component match the pre-arthritic tibial articular surface. The surgeon ensures the correct V–V orientation of the tibial resection with the knee in extension. After inserting a spacer block, the extension space should be tight and rectangular during a V–V laxity exam. The tibial resection’s correct F–E orientation, or posterior tibial slope, is verified by measuring the anteromedial and posteromedial thickness of the tibial resection. Any difference of a millimeter or more between the medial and lateral gaps for V–V balancing and anteromedial and posteromedial thicknesses for F–E balancing should be corrected [9,10]. The surgeon follows six options in a decision tree to set the V–V and posterior tibial slope orientations and thickness of the tibial component to restore the patient’s pre-arthritic tibial joint line and limb alignment and balance the knee, which in turn restores the native tibial compartment forces (Figure 2) [12,13,14,15]. The final step in balancing the KA TKA is choosing the optimal insert thickness that does not lead to over- or under-tension of the PCL, which is performed with the insert goniometer after inserting the trial components and is described below. 

### 2.2. Biomechanical Rationale for Using the Medial 1:1 Ball-in-Socket Insert with a Lateral Flat Surface

The sagittal plane conformity of the insert on the medial and lateral sides in conjunction with retention versus resection of the PCL determines axial plane tibiofemoral kinematics. Considering first the conformity of the medial side, medial-stabilized (MS) inserts generally fix the axis of I–E rotation in the medial compartment similar to the native knee, but inserts from different manufacturers have varying levels of conformity that affect internal tibial rotation in flexion [16,17,18]. A study of 21 patients undergoing caliper-verified KA TKA with PCL retention compared the degree of internal tibial rotation between medial 1:1 ball-in-socket inserts with less than 1:1 ball-in-socket conformity. Both inserts had an identical lateral flat articulation [19]. The mean tibial orientation for both insert conformities was similar in extension. In 90° and 120° flexion, however, the 1:1 ball-in-socket insert demonstrated 2° and 3° more internal tibial orientation, respectively. However, whether this minor increase in internal tibial orientation is clinically relevant remains to be seen.

Beyond increasing internal tibial rotation in flexion, the highly conforming medial 1:1 ball-in-socket insert confers stability in the A–P direction, similar to the native cruciate ligaments, and also helps preserve native tension of the PCL when retained [19]. By contrast, inserts with less than medial 1:1 ball-in-socket conformity allow the tibia to move anteriorly, thereby reducing the tension in the PCL that is required for achieving native internal tibial rotation in flexion. 

In addition to medial 1:1 ball-in-socket conformity, an equally important condition for restoring native knee kinematics is the sagittal plane geometry of the lateral surface of the insert. An insert with a lateral flat surface allows unconstrained tibial rotation and differs from posterior-stabilized (PS), cruciate-retaining (CR), and ultra-congruent (UC) insert geometries that have anterolateral and posterolateral rims, which cause a mechanical stop of tibial external rotation during knee extension and block internal tibial rotation during knee flexion, respectively. A study quantified the reduction in tibial rotation in passive motion caused by anterolateral and posterolateral rims. Two surgeons treated 23 patients undergoing KA with PCL retention [20] and determined the difference in passive tibial rotation between inserts with a lateral flat surface (F) and low-congruent (LC) and ultra-congruent (UC) inserts with anterolateral and posterolateral rims differentiated by an increase in millimetres of rim height. In comparison to the lateral flat surface, the LC and UC inserts resulted in a decrease in the magnitude of external tibial rotation in extension and internal tibial rotation at 90° flexion (Table 1). Thus, a medial 1:1 ball-in-socket insert with a lateral flat surface allows more natural internal and external tibial rotation. This axial plane motion is less likely with other insert geometries that have lateral concavities, where the rims function as “chock-blocks” [21]. 

Regarding in vivo weight bearing kinematics, a dynamic fluoroscopic study evaluated inserts with posterior-stabilized (PS) fixed bearings, ultra-congruent (UC) mobile bearings, and medial 1:1 ball-in-socket conformity with a lateral flat surface during gait activities such as level walking, downhill walking, and stair descent [22]. During stair descent, the insert with medial 1:1 ball-in-socket conformity and a lateral flat surface had the smallest range of A–P movement of the medial femoral condyle in all activities, along with the largest range of A–P movement of the lateral femoral condyle, which caused tibial internal–external rotation about a fixed medial pivot point in the medial compartment. A prospective, blinded, randomized controlled trial of gait motion with PS and CR inserts and an insert with medial 1:1 ball-in-socket conformity and a lateral flat surface found significantly less (approximately one-half) motion in a peak-to-peak anterior drawer and increased external rotation with the latter compared to the PS and CR inserts [23]. These findings confirmed that the axis of rotation of the KA TKA in the axial plane for the insert with medial 1:1 ball-in-socket conformity and a lateral flat surface was centered in the medial compartment like the native knee and shifted abnormally to the lateral compartment with the PS and CR inserts. Thus, the kinematics of the prosthetic knee for the insert with medial 1:1 ball-in-socket conformity and a lateral flat surface more closely matched those of the native knee not only in the sagittal plane but also in the axial plane. 

### 2.3. Biomechanical Rationale for Retaining the PCL 

Not only must the insert offer medial 1:1 ball-in-socket conformity and a lateral flat surface, but also the PCL must be retained to achieve native knee-like kinematics in the axial plane. In a cadaveric study, four surgeons performed a KA TKA using an insert with medial 1:1 ball-in-socket conformity and a lateral flat surface [24]. Before and after PCL excision, the I–E rotation of the tibia in extension and at 90° flexion in passive motion was compared. With retention of the PCL, the mean internal tibial rotation was 15°, whereas with excision of the PCL, the mean internal tibial rotation decreased to 7°. Likewise, in an in vivo study of weight-bearing deep knee bend, internal tibial rotation was determined for two groups of patients with and without PCL retention [25]. Mean internal tibial rotation with PCL retention was significantly greater at maximum flexion (18° vs. 10°). Collectively, these results regarding passive and weight-bearing flexion indicate that PCL tension drives internal tibial rotation and that retention of the PCL helps the prosthetic knee achieve mean internal tibial rotation of 18° comparable to the native knee [26,27]. Conversely, excision of the PCL caused a loss of about half of the internal tibial rotation from extension to 90° flexion. 

In addition to a loss of tibial rotation, a well-known consequence of resecting the PCL is an increase in flexion space laxity, which is inconsistent and unpredictable between knees [28,29,30]. Trying to compensate for this laxity by using a thicker insert or reducing the posterior slope does not correct the problem of internal tibial rotation loss caused by PCL excision [31]. 

### 2.4. Benefits of KA for Restoring Normal Patellar Tracking 

A by-product of restoring native knee internal tibial rotation in flexion is that the risk of patellofemoral complications is minimized contingent on the use of a femoral component with a trochlear design where the angle of the prosthetic trochlea is lateral to the quadriceps line of force [32]. Restoring internal tibial rotation in flexion reduces the Q-angle and maintains proper tension in the retinacular ligaments, which, in turn, ensures normal patellar tracking during knee flexion [24,33,34]. Thus, the screw-home mechanism of the native knee in extension and the natural internal tibial rotation in flexion is desirable in the prosthetic knee after a TKA.

## 3. The Goniometer Assists the Surgeon in Selecting the Optimal Insert Thickness, Which Is the Thickness That Simultaneously Provides the Greatest External Tibial Orientation in Extension and the Greatest Internal Tibial Orientation at 90° Flexion without Lift-Off of the Insert

### 3.1. Description and Illustration of the Insert Goniometer and Its Compatibility with an Insert with Medial 1:1 Ball-in-Socket Conformity and a Lateral Flat Surface

The intrinsic AP stability provided by a medial 1:1 ball-in-socket insert has eliminated the surgeon’s ability to select the optimal insert thickness by testing the A–P translation of the tibia relative to the femur using manual examination (drawer test) and to assess knee balance in the sagittal plane. Additionally, the surgeon should not rely on the force used to push a trial insert into position, as it was not useful to determine the correct thickness, supporting the necessity for a new objective measurement to make the selection, which the insert goniometer fulfills [35]. For the insert goniometer to function, it must provide medial 1:1 ball-in-socket conformity so that the I–E axis of rotation coincides with a medial pivot passing through the dwell point and a lateral flat surface without anterior and posterior rims to enable unrestricted I-E rotation about the medial pivot. With this native knee insert geometry, the insert goniometer can indirectly detect a loose and overtight PCL. Detecting an overtight PCL is necessary to reduce the risk of abnormal biomechanics, which can lead to a reduced range of movement, anterior subluxation of the tibia, an increased risk of tibial component subsidence, and polyethylene wear [5,36,37,38]. Detecting a loose PCL is necessary to reduce the risk of instability [6]. Hence, the insert goniometer was designed and validated for the surgeon to use as an intraoperative instrument as the final step of balancing during a KA TKA.

**Figure 3:**
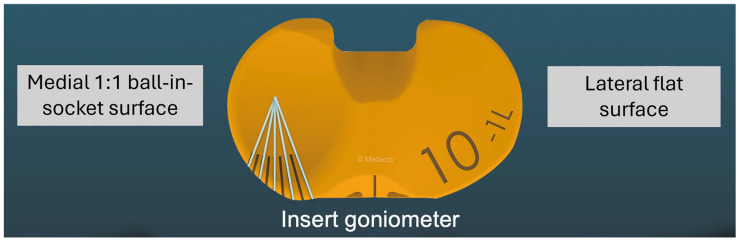
Image showing the design of the insert goniometer, which consists of lines in 5° increments that are calibrated to the axis of rotation or pivot point of the knee in the medial compartment. The lines extend over the anterior surface (see Figure 4) so that the I–E orientation of the tibia relative to the femur can be indicated.

**Figure 4:**
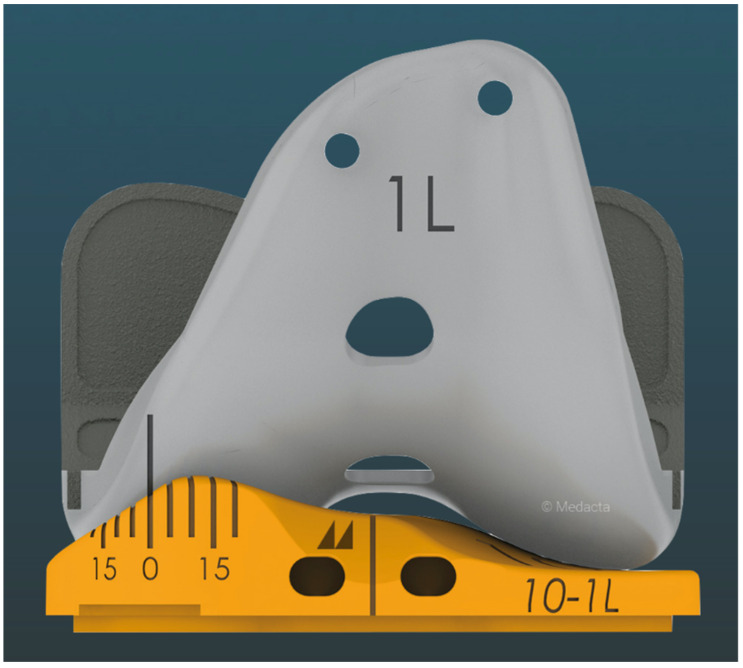
Image showing the method used by the insert goniometer to measure I–E orientation of the tibia relative to the femur. A reference line is laser etched vertically on the center of the medial condyle of the trial femoral component. As the tibia rotates internally/externally during flexion/extension, respectively, the I–E orientation is indicated. In this figure where the knee is extended, the indicated external orientation is 0°.

The design of the insert goniometer consists of markings in the front of the medial compartment of the insert that are calibrated to the axis of rotation, or pivot point of the tibiofemoral joint, which is the dwell point of the insert with medial 1:1 ball-in-socket conformity (Figure 3). For the insert goniometer to measure I-E tibial orientation, a reference line is laser etched vertically on the center of the medial condyle of the trial femoral component (Figure 4). Combining an angular scale on the medial insert and a vertical reference line on the medial femoral condyle enables the surgeon to measure external tibial orientation in extension and internal tibial orientation at 90° flexion intraoperatively during passive knee motion. 

Since its availability in August 2021, the insert goniometer has been used by the authors in all patients treated with a primary TKA. All patients underwent KA with PCL retention without restriction regarding the patient’s preoperative degree of flexion, varus and valgus deformity, and ligament condition. Up until July 2024, the experience has consisted of 2025 cases treated with an insert with 1:1 medial ball-in-socket conformity and a lateral flat articular surface; SMH performed 1097 of these procedures, and the insert goniometer’s designer (AJN) performed 928. Although the authors found that the insert goniometer was effective across a wide range of anatomical factors, its usefulness still needs to be assessed when the surgeon prefers to excise the PCL.

### 3.2. Example Describing and Illustrating the Use of the Insert Goniometer

**Figure 5:**
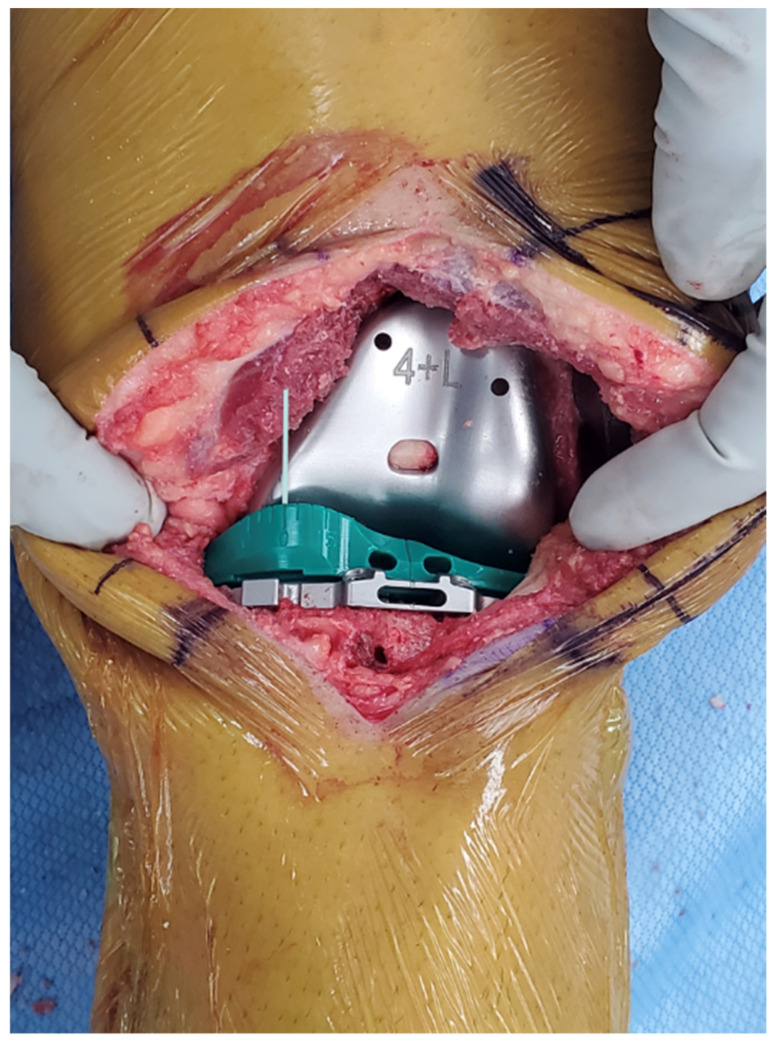
Photo of the insert goniometer at extension for an example case. The 10 mm thick insert goniometer had an external tibial orientation that measured 0°.

**Figure 6:**
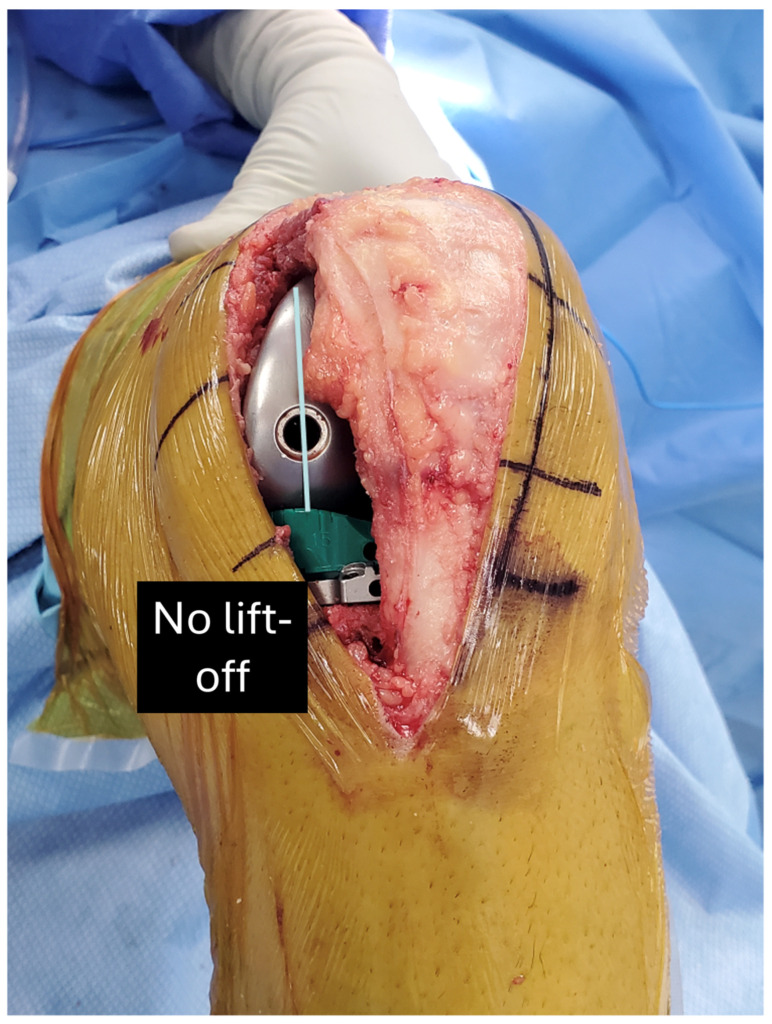
Photo of the insert goniometer at 90° flexion for an example case. There was no lift-off of the insert goniometer. The internal tibial orientation with the 10 mm thick insert goniometer was 10°, and there was no liftoff.

The following is a step-by-step example case to educate the interested surgeon on the use of the insert goniometer to select the optimal insert thickness. First, insert the trial femoral and tibial components. Place the insert goniometer with a thickness matching the spacer block, which, in this example, was 10 mm. Extend the knee by lifting the leg using the back of the hand without exerting an I–E axial moment to the ankle. Confirm that the knee fully extends and has negligible varus–valgus (V–V) laxity, indicating a tight rectangular space and verifying that the tibial component’s V–V orientation of the tibial baseplate resurfaces the pre-arthritic tibia. When the knee does not fully extend, either overcome a pre-existing flexion contracture by passively manipulating the knee into full extension or choose an insert goniometer that is 1 mm thinner. When the rectangular space is loose, choose an insert goniometer that is 1 mm thicker. Measure the external tibial orientation, which in this example was 0° external orientation for a 10 mm insert (Figure 5). Without applying an I–E rotational moment to the tibia, flex the knee to 90° and rest the foot on the operating table. Record the internal tibial orientation, which in this example was 10° for a 10 mm insert (Figure 6). Also, determine whether the insert’s anterior edge lifts off from the tibial baseplate. Anterior lift-off indicates a tight flexion space from too little posterior tibial slope, causing too much tension in the PCL. Remeasure the anteromedial and posteromedial thicknesses of the tibial resection. Correct the anterior lift-off by removing 1 or 2 mm from the posteromedial surface, which increases the tibial slope. 

Next, insert the 11 mm insert goniometer, which is 1 mm thicker. Confirm that the knee fully extends, and remeasure the external tibial orientation in extension. In this example, 2° was the external tibial orientation, which means that the tibia’s screw-home mechanism increased 2° compared to the 10 mm insert goniometer. Flex the knee to 90°, and confirm there is no lift-off of the insert goniometer. Measure the internal tibial orientation, which was 13° in this example, a 3° increase relative to the 10 mm insert goniometer. Since the external and internal orientations increased, the 11 mm thickness is more optimal than the 10 mm thickness.

**Figure 7:**
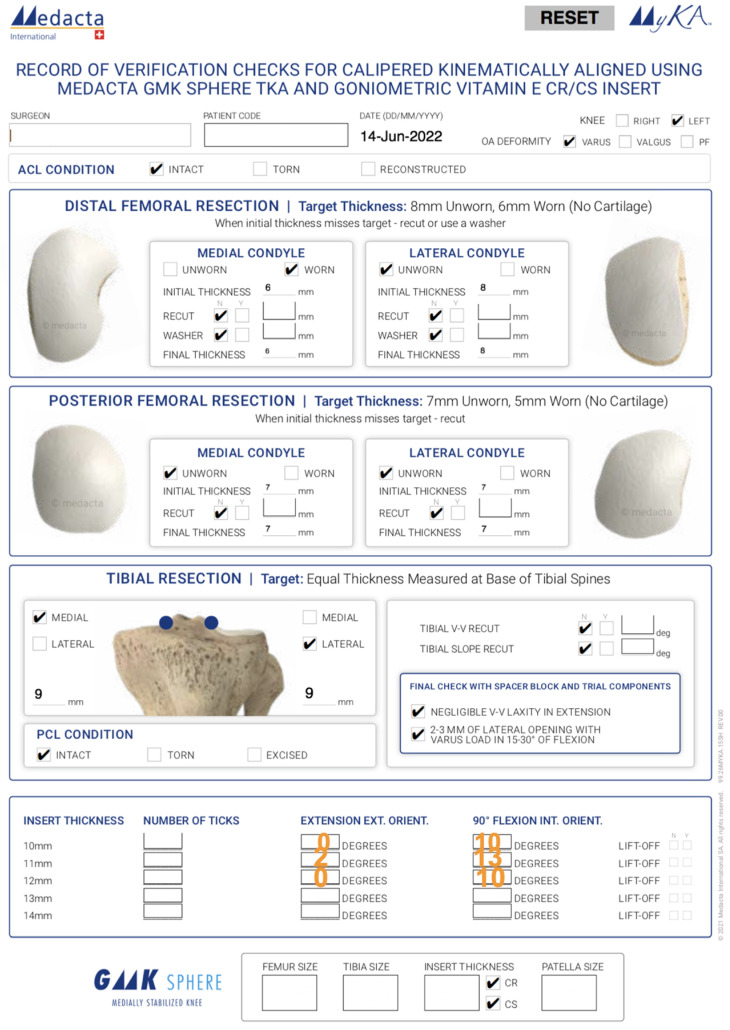
Verification check sheet for a caliper-verified KA TKA, which documents the thickness of the bony resections and the internal–external tibial orientations mentioned in the example case.

Next, position the 12 mm insert goniometer. In this example, the knee lost a few degrees of extension and the external orientation measured 0° (i.e., a loss of 2° of external orientation relative to the 11 mm insert goniometer). Flexing the knee to 90° resulted in a small amount of anterior lift-off of the insert goniometer, and the internal orientation measured 10°, indicating a loss of 3° of internal orientation. Consequently, the 11 mm thickness is optimal because of 2° more external tibial orientation and 3° more internal tibial orientation than the 10 mm and 12 mm thicknesses (Figure 7) [39]. 

## 4. Conclusions 

To restore native knee kinematics to the tibiofemoral joint, the surgeon needs to set the femoral and tibial components using KA and to use components that replicate the morphology of the native knee, which consists of an insert with medial 1:1 ball-in-socket conformity and a lateral flat surface without posterior and anterior rims. Restoring native external tibial orientation (i.e., screw-home mechanism) in extension and internal tibial orientation at 90° flexion necessitates that the surgeon leave the PCL intact. The final balancing step is to choose the optimal insert thickness using an insert goniometer, which the surgeon identifies as the thickness that enables full knee extension, maximum external tibial orientation (i.e., screw-home mechanism) in full extension, and maximum internal tibial orientation at 90° flexion without anterior lift-off of the insert relative to the tibial baseplate. 

## Figures and Tables

**Table 1:** Knee I–E rotation and incidence of anterior lift-off for three insert designs (+ external, − internal).

Knee Movement	Lateral Flat Surface	Low-Congruent Surface	Ultra-Congruent Surface
External orientation in extension	+9°	+5°	+2°
Internal orientation at 90° flexion	−13°	−9°	−7°
Mean total range	22°	14°	9°
Mean total loss	-	8°	13°
Anterior lift-off	0%	26%	57%

## Data Availability

Not applicable.
